# Mean and variability of QT-interval: Relevance to psychiatric illness and psychotropic medication

**DOI:** 10.4103/0019-5545.44898

**Published:** 2009

**Authors:** Rahul Kumar, Chaitra T. Ramachandraiah, Pratap Chokka, Vikram K. Yeragani

**Affiliations:** M.S. Ramaiah Medical College and Hospital, Bangalore, India; 1 Rajiv Gandhi University of Health Sciences, Bangalore, India; 2 Department of Psychiatry, University of Alberta, Edmonton, Alberta, Canada; 3 Department of Psychiatry and Behavioral Neurosciences, Wayne State University School of Medicine, Detroit, MI, USA; 4 Department of Biotechnology, Aacharya Nagarjuna University, A.P., India

The QT interval conceptually represents the duration from the onset of depolarization to the completion of repolarization of the cardiac cycle. On a standard ECG, it is measured from the beginning of the QRS complex (or RS complex if there is no Q wave) to the end of the T wave. This interval has been considered as a surrogate of action potential duration. The QT interval is slightly longer in women than in men, is affected by autonomic tone as well as catecholamines, and it shows circadian variations. The most important aspect of this interval is its relation with heart rate (the reciprocal of inter-beat-interval). A large number of formulae have been proposed to establish a rule allowing conversion of a pair of QT and R-R durations into a standardized QTc value corresponding to a “basal” R-R interval of 1 second. Bazett's formula is the most commonly used. Normally the QTc is between 350 to 430 milliseconds. Now there is substantial amount of evidence as to the electrophysiology and how changes in autonomic function can affect the function of the heart.[1–6]

The main myocardial cell type that determines the QT interval is the ‘M’ cell type. The cardiac action potential is generated by the changing transmembrane permeability to ion currents such as Na^+^, Ca^2+^ and K^+^. Like all living cells, the potential inside a myocyte is negative compared to the outside. However, cardiac cells are excitable and when appropriately stimulated, the ion channels within the cell membrane open and close sequentially. This changes the transmembrane ion permeability and leads to the sequential development of the transmembrane potential that is called the action potential. The initial depolarization (phase 0) is triggered by the rapid inward sodium (INa) and the L- and T-type calcium currents (ICa-L and ICa-T), which change the cell potential from -90 to 30 mV. The transient outward Ito potassium current is responsible for the slight repolarization immediately after the overshoot (phase 1). During the following plateau phase (phase 2), the cell potential is maintained by a balance between the inward L-type calcium current (ICa-L) and the electrogenic sodium-calcium exchange current (INaCa), and the outward Ito current. The repolarization phase (phase 3) of the myocyte is driven predominantly by outward movement of potassium ions, carried as the rapid (IKr) and slow (IKs) components of the delayed rectifier potassium current. The diastolic depolarization (phase 4) results from a combination of the decay of the outward delayed rectifier IKr and IKs currents, which maintains the resting potential at approximately -90 mV, and the activation of the inward pacemaker current (If) and the inward sodium background leak current (INa-B). A variety of other different potassium channel subtypes are also present in the heart.

Cardiac arrhythmias reflect disturbances of impulse initiation or impulse propagation. These consist of conduction blocks, reentrant rhythms and dysfunction of sino-atrial (SA) node and disturbances that originate from various ectopic foci. Paroxysmal ventricular tachycardia is the result of an ectopic focus in the ventricles and can be associated with bizarre QRS complexes in the ECG. This is much more serious than a paroxysmal supraventricular tachycardia as paroxysmal ventricular tachycardia could lead to ventricular fibrillation. Fibrillation is an irregular contraction and is ineffectual in propelling blood. In the ventricles, the vulnerable period coincides with the down slope of the T wave, and when a premature impulse arrives during the vulnerable period, this may lead to fibrillation. This may become self-sustaining due to reentrant process of cardiac excitability.

Tricyclic antidepressants (TCAs) have profound effects on cardiovascular system due to their strong autonomic effects and also a quinidine-like effect on cardiac conduction. This can lead to negative ionotropy, delayed intraventricular conduction, and a prolonged QT interval. Parenteral antipsychotics such as chlorpromazine or thioridazine can result in prolongation of the QT interval, and may result in Torsades de Pointes. It is also important to note that hypokalemia may be associated with acute schizophrenia, and hypokalemia can also prolong the QT interval. Even newer antipsychotics such as quetiapine and ziprasidone have been under scrutiny by the Federal Drug Administration authority in the USA (FDA). Thus, future studies need to systematically address this issue using other important measures such as QT dispersion and also QT variability.

## QT DISPERSION

Recently, QT dispersion has been used as a research and clinical tool to predict life-threatening arrhythmias.[7] In simple terms, QT dispersion is the difference between the longest and the shortest QT interval measured on the 12 lead ECG. These studies have reported greater dispersion of QT in patients who suffered sudden arrhythmic death than in a comparable group without arrhythmias in hypertrophic cardiomyopathy, long QT syndrome and in patients with chronic heart failure. Malignant arrhythmias are associated with increased heterogeneity of ventricular repolarization. This may also reflect in QT prolongation. However, QT prolongation may coexist without increased dispersion of ventricular repolarization. If ventricular repolarization is equally prolonged in all regions of the myocardium, a prolonged QT interval can occur with normal QT dispersion. On the other hand, an increased QT dispersion may occur on the background of a normal QT interval. Although QT dispersion has been shown to be more sensitive than the QTc in predicting serious arrhythmias, other studies are needed to make definitive conclusions. Some reports question the validity of this measure and there are few studies to evaluate the effects of psychotropics on QT dispersion.

## QT VARIABILITY

In a recent report, Berger and co-workers have described an algorithm to calculate QT intervals automatically from the digitized ECG and have shown that the QTvi, an index of QT variability, normalized for mean QT over HR variability, normalized for mean HR, was higher in symptomatic patients with dilated cardiomyopathy.[8] Atiga *et al.* have reported that QTvi was a better predictor of sudden cardiac death in cardiac patients compared to other measures such as ejection fraction, HR variability and ‘T’ wave alternans.[9] Now there are many other studies that have shown that QT variability is a valuable predictor of cardiac mortality. Thus, QTvi may be an important noninvasive tool to study certain populations at risk for cardiac mortality. Though, generally QT variability follows HR variability, there is not a complete coherence between these two time series.

We have recently shown that intravenous Isoproterenol and a change from supine to standing posture produce a highly significant increase in QTvi, thus linking it to an increase in sympathetic activity.[10] We have also found that pemoline and yohimbine, which are sympathomimetic agents, can increase QTvi, which links this index to an increase in sympathetic activity.[11,12] We have found that patients with panic disorder and depression have significantly increased QTvi compared to normal controls, which may be one of the factors responsible for the higher incidence of cardiovascular mortality in these patients.[13,14] Recently, we have also shown that there is a significant increase in QTvi in acute alcohol withdrawal and in unmedicated patients with schizophrenic illness.[15,16] This becomes very important in the context of increased cardiovascular morbidity during acute alcohol withdrawal and in patients with schizophrenia.

In another study, we found that nortriptyline, a TCA significantly increased QTvi in patients with panic disorder compared to paroxetine, an SSRI that had no significant effect on QTvi.[5] Both drugs were effective for the treatment of anxiety in these patients. Thus, choosing a drug with a safer cardiovascular profile is an important factor. Of particular importance is our recent report, suggesting a higher QTvi in children with anxiety disorders compared to normal children, though it was not accompanied by a decreased HR variability.[5]

Though QTvi still needs to be validated in future studies, it may prove valuable in the evaluation of cardiac side effects of a drug or in choosing a drug with an appropriate pharmacological profile in a given group of patients.

Pre-existing medical conditions such as congenital long QT syndrome, congenital heart disease, and hypothyroidism and serum electrolyte disturbances deserve special mention. As some psychotropics such as tricyclic antidepressants and antipsychotics, especially in higher doses can result in prolonged QT-interval, these should be used very cautiously in children with the above medical conditions.

Evaluation of the cardiovascular function is relatively simple and a routine clinical history and physical examination can identify conditions such as congenital heart disease, valvular disease, hypertension, and mitral valve prolapse. A 12-lead ECG can identify any abnormalities in QRS or QT intervals or bundle branch blocks. It is a good practice to have a baseline ECG for all children or adults before they receive any medication with appreciable effects on the cardiovascular system so that the effects can be monitored in a meaningful fashion. Finally, it is very important to stress the fact that there is a decrease in cardiac vagal function in conditions such as anxiety, depression, and schizophrenia and thus any drug that can increase sympathetic activity may have detrimental effects in these patients.
